# i-Tracker: For quantitative proteomics using iTRAQ™

**DOI:** 10.1186/1471-2164-6-145

**Published:** 2005-10-20

**Authors:** Ian P Shadforth, Tom PJ Dunkley, Kathryn S Lilley, Conrad Bessant

**Affiliations:** 1 Department of Analytical Science and Informatics, Cranfield University at Silsoe, Silsoe, Bedfordshire, UK; 2 Cambridge Centre for Proteomics, Biochemistry Department, Cambridge University, Cambridgeshire, UK

## Abstract

**Background:**

iTRAQ™ technology for protein quantitation using mass spectrometry is a recent, powerful means of determining relative protein levels in up to four samples simultaneously. Although protein identification of samples generated using iTRAQ may be carried out using any current identification software, the quantitation calculations have been restricted to the ProQuant software supplied by Applied Biosciences. i-Tracker software has been developed to extract reporter ion peak ratios from non-centroided tandem MS peak lists in a format easily linked to the results of protein identification tools such as Mascot and Sequest. Such functionality is currently not provided by ProQuant, which is restricted to matching quantitative information to the peptide identifications from Applied Biosciences' Interrogator™ software.

**Results:**

i-Tracker is shown to generate results that are consistent with those produced by ProQuant, thus validating both systems.

**Conclusion:**

i-Tracker allows quantitative information gained using the iTRAQ protocol to be linked with peptide identifications from popular tandem MS identification tools and hence is both a timely and useful tool for the proteomics community.

## Background

In recent years several techniques for protein quantitation by mass spectrometry have emerged. These include isotope-coded affinity tags (ICAT), metabolic labelling and stable isotope labelling of amino acids in culture (SILAC) [1-3]. These techniques enable peptides derived from two samples to be distinguished by mass spectrometry (MS). This is achieved though protein labelling with isotopically distinct tags (ICAT) or through the incorporation of isotopically distinct amino acids (SILAC), or a stable isotope labelled compound which represents the sole source of an element, typically nitrogen or carbon (metabolic label-ling). Protein quantitation can then be achieved by comparing the MS intensity of the peptides derived from the two samples. iTRAQ™ is a recently developed protein quantitation technique that utilizes four isobaric amine specific tags [4]. In single MS mode the differentially labelled versions of a peptide are indistinguishable. However, in tandem MS mode (in which peptides are isolated and fragmented) each tag generates a unique reporter ion. Protein quantitation is then achieved by comparing the intensities of the four reporter ions in the MSMS spectra.

The principal advantage of iTRAQ over ICAT, SILAC and metabolic labelling is that four samples can be analyzed simultaneously, thereby reducing the amount of mass spectrometry time needed for analysis. In addition, the b- and y- ions derived from peptides labelled with the four iTRAQ tags are indistinguishable, resulting in higher MSMS intensity and therefore more confident peptide identifications in comparison to ICAT, SILAC and metabolic labelling, in which the MSMS spectra for the differentially labelled peptides are acquired independently.

The ProQuant software, from Applied Biosciences (ABI), enables the quantitation and identification of iTRAQ labelled peptides. Peptide identification is achieved using ABI's Interrogator™ search algorithm. However, for the purpose of reporting iTRAQ results it is desirable to verify the proteins identified using a second, more widely used, MSMS search engine such as Mascot [5] or Sequest [6]. For this reason, the i-Tracker software has been developed to calculate iTRAQ reporter ion ratios and report them in a format that can be easily integrated with Mascot and Sequest search results.

i-Tracker takes as its input non-centroided mass spectra, either in the .dta format, as created by a program such as wiff2dta [7], or the .mgf files generated by the mascot.dll script for ABI's Analyst™ software. The software returns the relative ratios of each reporter ion. Indicative errors are provided to highlight the large discrepancies that may arise in the reported ratios when very low ion counts are used; they do not provide a model of all errors in the system, such as the probability if successful ion detection or counts introduced by background noise.

## Implementation

### Overview

The iTRAQ™ protocol uses four reporter ions of 114.1, 115.1, 116.1 and 117.1 Da. These are singly-charged and so found in the region 114 – 117 m/z in the mass spectra. Relative quantitation is performed by comparing the peak areas of each of these reporter ions in the mass spectrum. The default setting for i-Tracker assumes that the bulk of the peak will occur in the region of the reporter ion mass ± 0.05 Da. Each ion peak within this region is captured. The area of each reporter ion is then calculated by summing the areas of the trapezoids formed between each captured peak. This can be user-adjusted to suit the characteristics of the mass analyser used.

The reagents supplied by ABI are not 100% pure, but come with a datasheet by batch indicating the percentages of each reporter ion reagent that differ by -2, -1, +1 and +2 Da from the quoted mass. This quality control measure is taken into account by the i-Tracker software by adjusting each peak area as appropriate. The simultaneous equations needed for making this adjustment are solved using Cramer's rule. If the determinant of the initial matrix of coefficients is zero, or if no purity information is supplied, the software will output a warning and proceed without purity correction.

Following any purity correction, the peak areas are normalized. These normalized areas are used to calculate the quantitative ratios between each reporter ion. If the maximum peak height of any reporter ion is below the user-defined threshold for consideration, the string "UT" for under-threshold is the output. If there is no peak in the spectrum associated with a reporter ion as comparator (i.e. the denominator of the ratio calculation) the string "NA" is output.

Very low peaks in the mass spectra may suffer from large errors due to the quantized nature of the ion current. In order to provide some idea as to the potential magnitude of this error, a set of ratio-errors is reported which represent the maximum percentage error due to quantization. It should be noted that this reported error does not account for errors in detection of the ion current nor systematic error, such as background noise, in the measurement, but merely serves as a warning against placing too high confidence in reported ratios when these have been based on peaks with low ion counts.

### Detailed description

Items in this section are presented in the order in which data is processed by the software with one exception: The calculation for the determinant of coefficients, for purity correction, is performed very early in the processing sequence, in order to minimise repetitive calculation, whereas here it is presented as part of the purity correction section. Other than for this the following may be considered in parallel to the Perl code (i-Tracker.pl), which contains similar headings and flags for straightforward comparison.

#### Data input

Spectra must be non-centroided as the peak area calculations rely on the presence of all the peaks that would otherwise be combined in a centroided output.

i-Tracker can read spectra in .dta or .mgf formats. The two formats differ in the title information they contain and slightly in the format of the precursor ion information. However, the main difference in the way i-Tracker handles these files is that .dta files, which represent a single spectrum, are read into memory before processing whilst .mgf files are read in to memory spectrum by spectrum whilst keeping the input file open. Once a spectrum's information has been read, further processing is identical between input file formats.

#### Reporter ion peak collection

All peaks in the ranges:

114.1 ± 0.05

115.1 ± 0.05

116.1 ± 0.05

117.1 ± 0.05

are collected as a {mass}->intensity pair (hash). The default range of ± 0.05 was identified by considering the mass accuracy of the mass spectrometer and through manual inspection of a number of these peaks in the output files. This can be user-adjusted through an option presented at run-time.

#### Reporter ion area calculation

For each reporter ion peak range, the total area is calculated by summing the areas between ion peak pairs using the trapezoid approximation for calculating the area under a curve.

For example, a reporter ion peak may be comprised of four ions within the range considered. Here a, b, c and d are ion masses and a', b', c' and d' are their intensities. The total area (A) of this reporter ion peak is therefore:

A = (*b*-*a*) * 0.5 * (*a*'+*b*') + (*c*-*b*) * 0.5 * (*b*'+*c*') + (*d*-*c*) * 0.5 * (*c*'+*d*')

The maximum ion peak intensity is also identified at this point for comparison with the user-entered ion intensity threshold and for the calculation of quantisation errors.

#### Purity correction

Each batch of iTRAQ reagents supplied by ABI is labelled with sixteen purity values indicating the percentages of each reporter ion that have masses differing by -2, -1, +1 and +2 Da from the nominal reporter ion mass due to isotopic variants. This information can be used to correct the peak areas calculated for each reporter ion to account for the losses to, and gains from, other reporter ions. Losses to ion peaks not in the reporter ion range are also accounted for in this method.

The simultaneous equations needed to solve this problem are fairly complicated, but can be framed such that Cramer's rule may be applied. A detailed explanation of how to use Cramer's rule to solve simultaneous equations may be found in [8]. Briefly, if the determinant of the matrix of coefficients for the simultaneous equation is non-zero, the solution in each variable may be found. The four-way simultaneous equation for purity correction may be written as:

a,b,c,d,e,f,g,h,i,j,k,l,m,n,o,p are the sixteen purity correction values (as percentages) in the order:

114.1 – 2 Da, 115.1 – 2 Da, 116.1 – 2 Da, 117.1 – 2 Da, 114.1 – 1 Da, etc...

(NB This is a different logical order to that in which the user enters the values, they are rearranged within the program).

w,x,y,z represent the percentage of each peak expected to be present at the mass of the reporter ion associated with that peak. Here, w is for 114.1, x for 115.1 etc.:

w = (100 - (a + e + i + m))

x = (100 - (b + f + j + n))

y = (100 - (c + g + k + o))

z = (100 - (d + h + l + p))

The area (A_r_) of each reporter ion peak (_r_), as calculated above, can now be written in terms of the true areas of peaks (T_r_):



The task is now to calculate each T_r _according to these equations.

The determinant of the matrix of coefficients can be found:



If |C| is zero, then there is either an infinity of solutions or there are no solutions to these equations and so the purity correction module is skipped. If |C| is non-zero, purity correction proceeds:

The *Cramer determinants*, Δ_r_, are found to be:



The true areas, T_r_, can now be found:

T_r _= Δ_r_/|C|

#### Peak normalisation

Providing that the sum of the total areas is non-zero, normalised areas (N_r_) are calculated as:

N_r _= T_r _/ (T_114.1 _+ T_115.1 _+ T_116.1 _+ T_117.1_)

If the sum of all areas is zero, then each normalised area is also considered to be zero.

#### Under threshold checking

If the maximum ion peak intensity for any reporter ion peak area is equal to or less than the user-entered threshold, a flag of "UT" for "Under Threshold" is reported.

#### Ratio calculation

All ratios of true areas are calculated to three decimal places provided that the denominator is non-zero. If the denominator in any ratio calculation is zero, an "NA" flag is reported.

#### Quantisation error calculation

Very low ion counts may introduce a significant quantisation error. To some extent this is mitigated against by a sensible user-entered threshold of around 20 ion counts, but even so, comparing two reporter ion peaks that just pass such threshold could introduce an error of around 2.5% into the final ratios:

Eg. The user-entered ion count threshold is set to 19. The "correct" areas of peaks 1 and 2 should have been based on intensities of 20.5 and 19.5 respectively, but the reported ratio is 1:1 due to the quantum nature of ion counts. A quantisation error of 2.5% has been introduced in this case. For ion counts lower than this, the potential quantisation error will be much greater, but their ratios in this case would have been masked by the user-entered threshold.

These potential quantisation errors are reported alongside the peak ratios to highlight instances where results might be compromised by this effect. They are calculated as a percentage error between two ratios thus:

Err(1,2) = (100 * ((0.5 / Peak1Max) + (0.5 / Peak2Max))

these are output in the errors matrix for each ratio.

Similar potential quantisation errors in the normalised areas are calculated as:

Err(1) = (100 * (0.5 * Peak1Max))

these are output in the left-right diagonal of the quantisation errors matrix.

### Output format

Results are output in a choice of two comma-separated-variable (CSV) formats readily imported into R, Excel and other packages. One of these is designed for human-readability whereas the other is more convenient for simple analysis in a spreadsheet. Both are simple to parse for more detailed analysis. Full descriptions of both formats are provided in the user-instructions. The title information for each spectrum's results is taken from that of the input spectrum and hence matching of quantitative data with peptide identifications is straightforward. Each set of ratios is reported by spectrum, in the order in which they appear in either the directory or the input file (depending on .dta or .mgf inputs). Linking iTRAQ ratios, as determined by i-Tracker, and peptide identifications is dependent on the user being able to accurately link the identified spectra to this output data. Either the filename, for .dta input, or the Mascot peptide "Title" information can be used for this purpose, as performed in the GAPP project prototype .

## Results and discussion

**Figure 1:**
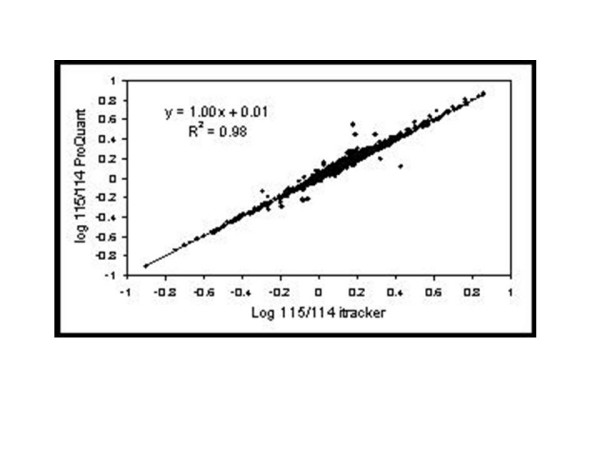
**Comparison of ProQuant and i-Tracker results**. Log ratios of reporter ions 115:114 are shown for ProQuant and i-Tracker. The user-entered intensity threshold for i-Tracker processing was set to 20 ion-counts.

Relative quantitation data using i-Tracker were compared to the output from ProQuant and found to be in good agreement, as shown in Figure 1. The data used were derived from Arabidopsis membrane protein samples labeled with iTRAQ reagents. Arabidopsis membrane protein samples were prepared as described in [9]. Membrane pellets were solubilised in 100 μl of labelling buffer (50 mM TEAB, 8 M Urea, 2 % Triton X-100 and 0.1 % SDS). 100 μg of protein were reduced with 5 mM TCEP for 1 h at 20°C and cysteines were blocked with 10 mM MMTS for 10 min at 20°C. Samples were then diluted with 50 mM TEAB, in order to reduce the urea concentration to 1 M. 5 μg trypsin were added to the samples, which were then incubated for 15 h at 37°C. The peptide samples were then labelled with the iTRAQ reagents as described in [4].

The pooled labelled peptides were loaded onto a Dionex ProPac SCX-10 strong cation exchange column (250 mm × 2 mm i.d.) at 0.3 ml/min and separated using a gradient of 0 mM to 500 mM NaCl over 50 min during which time 17 fractions were collected and analyzed by LC-MSMS using an ABI QSTAR mass spectrometer.

The data reported in Figure 1 represent one of these 17 fractions, picked at random and processed using i-Tracker and ProQuant. i-Tracker was run using an ion count threshold of 20 and the appropriate purity correction factors as supplied by ABI. The ratios of the two reporter ions shown (114 and 115) range from 0.12 to 7.3. Other comparisons, not shown here, demonstrate similar performance. These results demonstrate that the output from i-Tracker is almost identical to that of ProQuant in terms of calculating the ratios of reporter ion peaks. As ProQuant was developed by ABI, this very high correlation is desirable, but it should be noted was not part of the original specification for i-Tracker. If the results had been markedly different, there would have been a question to answer as to the validity of the results from both systems. As i-Tracker was developed entirely independently from the developers of ProQuant, with no information as to ABI's algorithm being sought or provided, the convergence of the end results provides a positive validation of either a common sense of the design or of the implementation of both systems. The first of these assertions would apply if the algorithms are independently identical. In this case similar design decisions would have been made when presented with the same problem, leading to a corroborative validation of the design. On the other hand, if the algorithms are different, then the results provide a demonstration that both algorithms perform to the same specification. Which of these is the true position is unknown as ABI have as yet not released details of their ProQuant algorithm. This also prevents a complete analysis of the very few outliers, around 5 out of 1463 data points present in the comparison.

A current limitation of i-Tracker is that it only accepts two types of input file; the .dta and .mgf formats. Although a number of converters are publicly available, it would be beneficial for i-Tracker to be modified such that it can handle the more generic MS file types available, such as mzXML and mzData. The advantage of using these would be that they are set to become standard across the community. However, as both are XML-based and contain encoded peak lists, processing these is more complicated than for the file-types currently handled. This extension to i-Tracker will be addressed in future releases.

## Conclusion

i-Tracker provides quantitative proteomic information for peptides when using the iTRAQ reagents supplied by ABI. The principal advantage to using i-Tracker is that the results are provided in a form that may be easily linked to peptide identifications made using software other than that provided by ABI, something which is currently time-consuming and difficult using ProQuant. Furthermore, both the algorithm and source code for i-Tracker are freely available and therefore may be reviewed and developed further by the proteomics community.

## Authors' contributions

CB, KL, TD and IS together conceived and developed the initial specification for the tool. IS designed and built the i-Tracker application with extensive input in the design and user-requirements from TD. TD carried out the sample collection, mass spectrometry and data analysis. IS drafted the manuscript with input from TD, KL and CB. All authors read and approved the final manuscript.

## Availability and requirements

**Project name**: i-Tracker

**Project home page**:  The software may be freely downloaded from this website. As it is provided as a Perl script, the source code is naturally available.

**Operating system(s)**: Platform independent. A Windows (XP) executable is provided as well as the Perl script.

**Programming language**: Perl

**Other requirements**: The freely available Perl modules Time::localtime and Math::MatrixReal are required unless using the Windows executable.

**License**: GNU GPL

**Any restrictions for use by non-academics**: None.

## References

[B1] Gygi SP, Rist B, Gerber SA, Turecek F, Gelb MH, Aebersold R (1999). Quantitative analysis of complex protein mixtures using isotope-coded affinity tags. Nature Biotechnology.

[B2] Krijgsveld J, Ketting RF, Mahmoudi T, Johansen J, Artal-Sanz M, Verrijzer CP, Plasterk RHA, Heck AJR (2003). Metabolic labeling of C-elegans and D-melanogaster for quantitative proteomics. Nature Biotechnology.

[B3] Ong SE, Blagoev B, Kratchmarova I, Kristensen DB, Steen H, Pandey A, Mann M (2002). Stable isotope labeling by amino acids in cell culture, SILAC, as a simple and accurate approach to expression proteomics. Molecular & Cellular Proteomics.

[B4] Ross PL, Huang YLN, Marchese JN, Williamson B, Parker K, Hattan S, Khainovski N, Pillai S, Dey S, Daniels S, Purkayastha S, Juhasz P, Martin S, Bartlet-Jones M, He F, Jacobson A, Pappin DJ (2004). Multiplexed protein quantitation in Saccharomyces cerevisiae using amine-reactive isobaric tagging reagents. Molecular & Cellular Proteomics.

[B5] Perkins DN, Pappin DJC, Creasy DM, Cottrell JS (1999). Probability-based protein identification by searching sequence databases using mass spectrometry data. Electrophoresis.

[B6] Eng JK, McCormack AL, Yates III (1994). An approach to correlate tandem mass spectral data of peptides with amino acid sequences in a protein database. Journal of the American Society for Mass Spectrometry.

[B7] Boehm A, Galvin R, Sickmann A (2004). Extractor for ESI quadrupole TOF tandem MS data enabled for high throughput batch processing. BMC Bioinformatics.

[B8] Riley KF, Hobson MP, Bence SJ (1998). Mathematical Methods for Physics and Engineering.

[B9] Dunkley TP, Watson R, Griffin JL, Lilley KS (2004). Localization of organelle proteins by isotope tagging (LOPIT).. Molecular Cell Proteomics.

